# Novel Approach for Insertion of Heterologous Sequences into Full-Length ZIKV Genome Results in Superior Level of Gene Expression and Insert Stability

**DOI:** 10.3390/v12010061

**Published:** 2020-01-03

**Authors:** Evgeniya Volkova, Konstantin A. Tsetsarkin, Emilia Sippert, Felipe Assis, Guangping Liu, Maria Rios, Alexander G. Pletnev

**Affiliations:** 1 Office of Blood Research and Review, Center for Biologics Evaluation and Research, Food and Drug Administration (FDA), Silver Spring, MD 20993, USA; evgeniya.volkova@fda.hhs.gov (E.V.); emilia.sippert@fda.hhs.gov (E.S.); felipe.assis@fda.hhs.gov (F.A.); 2 Laboratory of Infectious Diseases, National Institute of Allergy and Infectious Diseases (NIAID), National Institutes of Health (NIH), Bethesda, MD 20892, USA; konstantin.tsetsarkin@nih.gov (K.A.T.); guangping.liu@nih.gov (G.L.)

**Keywords:** Zika virus, flaviviruses, heterologous gene expression, bioluminescence, reverse genetics

## Abstract

Zika virus (ZIKV) emerged in the Americas in 2015, presenting unique challenges to public health. Unlike other arboviruses of the *Flaviviridae* family, it is transmissible by sexual contact, which facilitates the spread of the virus into new geographic areas. Additionally, ZIKV can be transmitted from mother to fetus, causing microcephaly and other severe developmental abnormalities. Reliable and easy-to-work-with clones of ZIKV expressing heterologous genes will significantly facilitate studies aimed at understanding the virus pathogenesis and tissue tropism. Here, we developed and characterized two novel approaches for expression of heterologous genes of interest in the context of full-length ZIKV genome and compared them to two previously published strategies for ZIKV-mediated gene expression. We demonstrated that among the four tested viruses expressing nLuc gene, the virus constructed using a newly developed approach of partial capsid gene duplication (PCGD) attained the highest titer in Vero cells and resulted in the highest level of nLuc expression. Suitability of the PCGD approach for expression of different genes of interest was validated by replacing nLuc sequence with that of eGFP gene. The generated constructs were further characterized in cell culture. Potential applications of ZIKV clones stably expressing heterologous genes include development of detection assays, antivirals, therapeutics, live imaging systems, and vaccines.

## 1. Introduction

ZIKV is a mosquito-borne virus from the *Flaviviridae* family, genus *Flavivirus*. Since its discovery in 1947 in Uganda, the virus has remained relatively dormant throughout the most of the 20th century. However, in the first decade of the 21st century, ZIKV reemerged in a series of large-scale outbreaks on several islands in the Pacific Ocean. Subsequently, the virus reached continental Americas in 2015, where it caused the largest-to-date outbreak in documented history [1]. It was shown that while in most cases ZIKV infection results in self-limiting febrile illness, some patients present with neurological complications such as Guillain-Barré syndrome, and infection during pregnancy may cause microcephaly and other severe developmental abnormalities of the fetus (Reviewed in [2]). The follow-up studies of the apparently unaffected babies born from the mothers who contracted ZIKV during pregnancy also indicated a high risk for development of neurological abnormalities later in life (reviewed in [3,4]). This information, coupled with the fact that ZIKV is able to be transmitted through non-vector routes of infection (sexual, intrauterine, perinatal), makes research of ZIKV pathogenesis and development of vaccines, antiviral therapeutics, and sensitive and specific assays for detection of the virus a high priority [5,6].

Viral genomes engineered for foreign gene expression can be a valuable tool in gene therapy, drug delivery, and vaccine development studies [7,8,9,10]. Viruses expressing heterologous gene(s) of interest can be utilized in high-throughput screening of virus inhibitor compounds or cell-based antiviral effectors and in viral detection tests. Moreover, studies aimed at evaluation of efficacy of vaccines or antivirals can benefit from using in vivo imaging of live animals, which allows monitoring temporal and spatial progression of viral replication [11], where minimizing the differences between the individual animals using non-invasive techniques are preferable.

Like all flaviviruses, ZIKV has a positive sense, single-stranded RNA genome approximately 11 kb in length. The genome contains 5′ and 3′ untranslated regions and encodes a single polyprotein, which is processed co- and post-translationally into three structural and seven non-structural proteins [12]. Because of the relatively small genome size and the monocistronic type of genome organization, the development of replication-competent recombinant flaviviruses stably expressing heterologous genes of interest in cell culture or in vivo has proved challenging [13,14,15,16]. Prior to this study, two approaches to generate replication-competent recombinant ZIKV expressing reporter genes had been described [17,18], both of which were based on the previously published strategy of capsid gene duplication [19]; however different ZIKV strains and different reporter genes were used, which would not allow us to perform a direct comparison of the level of heterologous gene expression and insert stability between these two approaches. Moreover, the criteria for selection of the particular portion of the C gene used for gene duplication and the verification of optimal genetic configuration of C gene regulatory region were not provided. The appropriate configuration is required for efficient ZIKV replication, and if not verified may have negative impact on the insert’s stability and on the levels of heterologous gene expression, especially during virus replication in complex biological systems such as live animals [13].

To overcome these limitations, we characterized a complete C gene regulatory region of ZIKV and used this knowledge to construct a full-length ZIKV capable of heterologous gene expression in the duplicated capsid gene region (dCGR). In addition, we adapted for ZIKV an approach for expression of heterologous genes in the duplicated E/NS1 region of flaviviruses, which is similar to that described earlier for yellow fever 17D and Langat viruses [20,21]. To perform side-by-side comparison of newly developed and previously published approaches for efficacy of/suitability for heterologous gene expression, we constructed four replication-competent infectious clones all carrying the nanoLuciferase (nLuc) gene in the genetic background of the same strain of ZIKV and analyzed their replicational fitness, stability, and level of heterologous genes expression in Vero cells. The luciferase-based system was chosen due to superior sensitivity of enzymatic versus non-enzymatic platform for gene detection. We showed that the dCGR, but not the E/NS1 region, is the most suitable site in the ZIKV genome for heterologous gene insertion and that the combination of codon optimization and frame shifting mutations is necessary and sufficient to prevent the ejection of the reporter gene during prolonged cell passaging. We further characterized the constructs created based on the dCGR approach in different cell lines relevant to ZIKV pathogenesis and evaluated stability of the viruses during consecutive passaging.

## 2. Materials and Methods

### 2.1. Cell Culture Systems

BeWo (human placenta; ATCC#CCL-98) cells were maintained in DMEM, high glucose, GlutaMAX™ Supplement, pyruvate (ThermoFisher Scientific, Waltham, MA, USA) additionally supplemented with 10% fetal bovine serum (FBS) (Atlanta Biologicals, Flowery Branch, GA, USA) and 1% penicillin/streptomycin solution (ThermoFisher Scientific) at 37 °C and 5% CO_2_. JEG-3 (human placenta; ATCC#HTB-36) cells were maintained in MEM (ThermoFisher Scientific) containing 10% FBS, 2 mM l-Glutamine, and 1% penicillin/streptomycin solution at 37 °C and 5% CO_2_. Hep G2 (human liver cancer; ATCC#HB-8065) cells were maintained in DMEM containing 10% FBS, 2 mM l-Glutamine, and 1% penicillin/streptomycin solution at 37 °C and 5% CO_2_. Monocyte-derived macrophages (MDM) were produced by culturing elutriated monocytes from a blood donor in 25 cm^2^ flasks in complete RPMI-1640 media (ThermoFisher Scientific) supplemented with 10% FBS, 2 mM l-Glutamine (Mediatech, Manassas, VA, USA), macrophage colony-stimulating factor (M-CSF) (Sigma-Aldrich, St. Louis, MO, USA) at 10 µg/mL, and 1% penicillin/streptomycin solution at 37 °C and 5% CO_2_ for 2 weeks. Adherent mosquito C6/36 (*Aedes albopictus;* ATCC#CRL-1660) cells were maintained in MEM supplemented with 10% FBS, 2 mM l-Glutamine, and 1% penicillin/streptomycin solution at 32 °C and 5% CO_2_. LLC-MK2 (Rhesus monkey kidney; ATCC#CCL-7) cells were maintained in MEM supplemented with 5% FBS, 2 mM l-Glutamine, and 1% penicillin/streptomycin solution at 37 °C and 5% CO_2_, and WHO Vero (African green monkey kidney) were maintained either in the same media used for LLC-MK2 or in OptiPRO-SFM (ThermoFisher Scientific) supplemented with 2 mM l-Glutamine and 1% penicillin/streptomycin. Human monocytes were obtained under informed consent (FDA IRB-approved protocol #03-120B, initially approved in 2003).

### 2.2. Construction of Infectious ZIKV Clones

**Figure 1:**
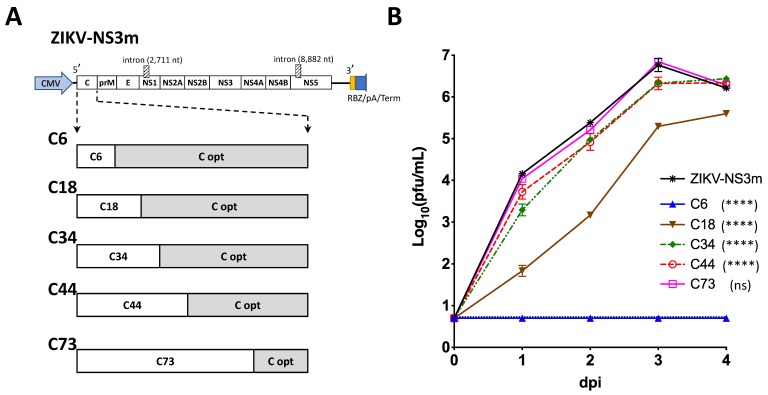
Mapping the region containing regulatory elements of the C gene of ZIKV. (**A**) Schematic representation of ZIKV-NS3m infectious clone (on top of (**A**)), which was used to construct a panel of viruses with chimeric C gene sequence (on the bottom of (**A**)). White boxes represent wt sequence of ZIKV-NS3m. Gray boxes (C opt) represent sequences that were mutated by synonymous substitutions. (**B**) Growth kinetics of viruses with chimeric C gene sequence in Vero cells after plasmid DNA transfection. Mean viral titer ± standard deviations in the samples that were collected daily from duplicate flasks were determined by titration in Vero cells. Dotted line (the one right above the blue C6 line) represents limit of virus detection (0.7 log_10_ pfu/mL). Differences between growth of ZIKV-NS3m and that of the other constructs were compared using two-way ANOVA (**** *p* < 0.0001; ns—not significant, *p* > 0.05).

Construction of all clones produced for this study was carried out using standard and advanced PCR-based cloning techniques [22]. Infectious clone of the Paraiba_01/2015 strain of ZIKV (ZIKV-ICD) and Vero cells adapted version of this strain (ZIKV-NS3m) were described elsewhere [23]. In both clones, the full-length ZIKV cDNA genome is transcribed from cytomegalovirus (CMV) promoter by host cell RNA polymerase II (Figure 1A). Transcription termination is ensured by RNA polymerase II terminator sequence located downstream of the genome, preceded by SV40 early polyadenylation signal, and the precise cleavage of 3′ end of the ZIKV RNA is performed by the hepatitis delta virus (HDV) ribozyme.

To produce infectious clones (ic) carrying heterologous sequences, the insertions were made in ZIKV C protein gene region as reported earlier or E/NS1 region using conventional PCR-based cloning techniques [17,18,19,24]. Sequences encoding nLuc and enhanced green fluorescent protein (eGFP) genes were amplified from plasmids pNL1.1 (Promega) or pEGFP, respectively. To improve the stability of the produced infectious clones in bacteria, additional intron sequences were introduced into the first C gene sequence of nLuc-50C/FrSh, nLuc-fullC, and into the first C gene and eGFP gene sequences of GFP-50C/FrSh constructs (for schematic representation of intron locations, see Figure S1A). The foot-and-mouth disease virus (FMDV) 2A protease sequence was amplified from plasmid T/1674-mirV2 [25]. Ubiquitin sequence (nts 1-228 in GenBank # EU249809.1) and codon optimized sequences of different region of ZIKV genome were synthesized by Integrated DNA Technologies (Coralville, IA, USA). Ubiquitin and 2A protease were used to ensure proper processing of proteins. Cloning strategies and plasmid sequences are available upon request. Large-scale plasmid preparations were produced in *E. coli* (strain MC1061) in LB media with shaking overnight at 37 °C, and plasmid DNA was purified using EndoFree Plasmid Maxi Kit (Qiagen, Hilden, Germany). To verify genetic integrity, we sequenced all regions of the final plasmids that were generated for cloning using PCR technique. In addition, regions of plasmid DNA containing reporter genes were PCR amplified, and plasmids were subjected to restriction analysis (Figure S1B,C).

### 2.3. Virus

ZIKV strain Paraiba_01/2015 (GenBank #KX280026) was kindly provided by Steven Whitehead, NIAID, NIH. It was initially isolated from serum of a febrile patient in Brazil and had been passaged twice in C6/36 cells and twice in Vero cells.

### 2.4. DNA Transfections

WHO Vero cells were seeded onto 12.5 cm^2^ flasks or 24-well plates in DMEM containing 10% FBS a day before transfection. Two hours prior to the procedure, media was replaced with Opti-MEM Reduced Serum Media (ThermoFisher Scientific). Lipofectamine 2000 (ThermoFisher Scientific) was used for transfection of the cells with 2.5 µg (1 µg for 24-well plates) of each infectious DNA clone following manufacturer’s instructions. Cells were incubated for 4 h at 37 °C and 5% CO_2_ with periodic agitation of media, washed with DMEM containing 10% FBS, and maintained in this media at the same conditions for 4–5 days.

### 2.5. Plaque-Forming and Focus-Forming Assays

The virus titer was determined by either plaque-forming assay (PFA) or focus-forming assay (FFA), depending on the ability of the virus to cause cytopathic effect (CPE). PFAs were performed for Paraiba_01/2015 and ZIKV ic, and FFAs were performed for GFP-50C/FrSh and nLuc-50C/FrSh. Briefly, WHO Vero cells were seeded in 24-well plates to reach 80–100% confluence on the day of the assay. Virus samples were ten-fold serially diluted in MEM supplemented with 1% FBS, and 100 µL of each dilution were used to infect the cells in duplicates. After 1 h incubation at 37 °C and 5% CO_2_ with periodic rocking of the plates, cells were overlaid with 0.8% methylcellulose in DMEM containing 2% FBS and 2 mM l-Glutamine and maintained at 37 °C and 5% CO_2_ for 4–5 days. Cells were fixed with cold methanol, and either stained with crystal violet for PFA or subjected to immunostaining using Anti-Flavivirus Group Antigen Antibody 4G2 (EMD Millipore, Burlington, MA, USA), goat anti-mouse HRP-conjugated secondary antibody (Jackson Immuno, West Grove, PA, USA) and True Blue peroxidase substrate (KPL, Silver Spring, MD, USA) for FFA.

### 2.6. Infections

Cells were seeded onto appropriate vessels (6-well plates, 25 cm^2^ flasks for MDMs, or 24-well plates for cells infected with nLuc-50C/FrSh) 24 h prior to infection. On the day of infection, cells in sub-confluent monolayers were counted with Countess automated cell counter (ThermoFisher Scientific) and infected with viruses at a multiplicity of infection (MOI) of 0.01 in cell-specific base media containing 1% FBS (except for MDMs, for which growth media was used). Infection was performed in duplicates for 1 h at 37 °C with periodic shaking, after which inoculums were removed, and cells were washed twice with appropriate complete media and overlaid with the same media before incubation at cell-specific conditions for the desired amount of time.

### 2.7. Viral Replication Kinetics

Following transfection or infection, cells were washed twice with appropriate media, and 300 µL aliquots of cell supernatants were harvested daily. The media volume in the well or flask was replenished with the same amount of corresponding media. Media from nLuc-50C/FrSh infected or transfected cells (except MDMs) was harvested from two designated wells of 24-well plates at appropriate time points, and luciferase readings were performed as described below. FFA was used to determine infectious titers. To identify statistically significant differences in viruses’ growth kinetics, two-way ANOVA with multiple comparisons tests with Tukey corrections was performed using GraphPad Prism software version 6 (San Diego, CA, USA). *p* values ≤ 0.05 were considered significant.

### 2.8. eGFP and Luciferase Reporter Assays

Cells were infected with GFP-50C/FrSh or transfected with plasmids GFP-50C/FrSh or GFP-E/NS1. At different time points, eGFP expression was observed under a fluorescent microscope (Olympus IX71, Olympus, Tokyo, Japan) and documented. For nLuc expression quantification, nano-glo luciferase assay system (Promega, Madison, WI, USA) was used according to manufacturer’s instructions. Briefly, after equilibrating all reagents to room temperature and removal of media, fresh media was added to the well/flask, and equal volume of nano-glo lysis buffer containing luciferase substrate (1:50) was added. In 3 min, the media/buffer solution was pipetted up and down several times and transferred to an Eppendorf tube. In 10 min after addition of buffer, a preliminary measurement was taken on TD-20/20 Luminometer (Promega) to evaluate the levels of luciferase. If needed, 1:10, 1:100, or 1:1000 dilutions were prepared in MEM supplemented with 5% FBS, 2 mM l-Glutamine, and 1% penicillin/streptomycin solution. Final luciferase reading was performed 20–60 min after buffer/substrate addition. Alternatively, readings were performed in duplicates in white 96-well polystyrene plates using Victor X3 multilabel plate reader (Perkin Elmer, Waltham, MA, USA) or SpectraMax M Series multi-mode microplate reader (Molecular Devices, San Jose, CA, USA).

### 2.9. Reporter Virus Stability Assessment

To determine whether the infectious clone-derived viruses can sustainably carry the reporter genes, GFP-50C/FrSh and nLuc-50C/FrSh viruses were passaged ten times in Vero cells. To initiate serial passaging, viruses recovered after pDNA transfection were used to infect Vero cells in duplicate 25 cm^2^ flasks of Vero cells at an MOI of 0.1, and cell culture supernatants were collected on day 3 or 4 post-infection (dpi). For infection of each subsequent passage, 20 µL of supernatant from the previous passage was used to infect Vero cells in 25 cm^2^ flasks. The eGFP and nLuc expression and virus titers were assessed at multiple time points during passaging. Parts of viruses containing the reporter gene inserts were sequenced.

## 3. Results

### 3.1. Mapping the Regulatory Elements That Constitute Replication Promoter of the C Gene of ZIKV

Apart from encoding amino acid sequence of capsid (C) protein, the C gene of the flaviviruses contains various regulatory elements critical for virus replication. These include the cyclization sequence, which participates in interaction with 3′ terminus of genomic RNA, as well as additional auxiliary *cis*-acting elements regulating virus RNA synthesis and translation ([26,27,28], reviewed in [29]). The structure and composition of the regulatory elements of the C gene of ZIKV has yet to be reported. Since the objective of this study was to develop ZIKV clones expressing gene(s) of interest in the region of capsid gene duplication, we began our analysis by mapping the region containing regulatory elements of the C gene of ZIKV.

**Figure 2:**
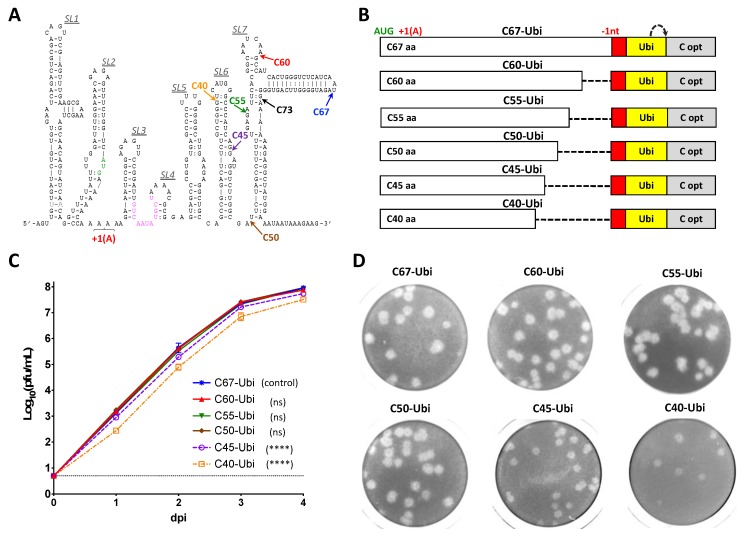
Identification of a minimal region of C gene that is required for ZIKV growth in Vero cells by deletional analysis. (**A**) Predicted stem-loop structure of the 5′-end of ZIKV genome (strain Paraiba_01/2015). Colored arrows indicate codon positions at which 5′ terminus of wt C gene sequences were fused with target sequence for mir-124 (red box in panel B). Sequence highlighted in green indicates translation initiation codon AUG of ZIKV polyprotein. Sequence highlighted in magenta indicates 5′ genome cyclization sequence. +1 (A) shows the position of ORF-shifting insertion (+1 nt) of a single A residue. (**B**) Schematic representation of viral genomes featuring gradual reduction of the wt C gene sequence from the 3′-end. Red and yellow boxes indicate target for mir-124-3p and ubiquitin gene, respectively. Gray boxes (C opt) represent full-length sequences of C gene that were mutated by synonymous substitutions. (+1 A) and (-1nt) are positions of ORF shifting and ORF restoring sequence modifications, respectively. Dotted arrow represents the site of cleavage by ubiquitin. (**C**) Growth kinetics of viruses in Vero cells after plasmid DNA transfection. Differences between growth kinetics of C67-Ubi (mean virus titers for dpi 1–4) and those of the other five viruses depicted in panel B were compared using two-way ANOVA (**** *p* < 0.0001; ns—denotes not significant, *p* > 0.05). Dotted line represents limit of virus detection (0.7 log_10_ pfu/mL). (**D**) Plaque morphology of viruses in Vero cell monolayer. Infected cells in 24-well plates were fixed at 5 dpi, and viral plaques were visualized by crystal violet staining.

To map the region containing *cis*-acting elements of C gene, we generated a panel of ZIKV clones with chimeric C gene sequences (Figure 1A). Each of the clones contained intact wild type (wt) C gene sequence of the ZIKV-NS3m virus at the 5′-terminus of the gene, while the 3′-terminus was mutated by synonymous substitutions in each of AA codons (C-opt sequence). Infections clone ZIKV-NS3m has been reported earlier [23]. Placement of the junction site between C wt and C-opt sequences at various locations allows mapping of the minimal region sufficient for ZIKV replication. Clones with chimeric C gene sequences were transfected into Vero cells and kinetics of virus recovery were compared to those of the parental ZIKV-NS3m virus. Preserving 219 nucleotides (nts) of the wt C gene (encoding 73 AA) did not cause detectable attenuation of C73 virus replication in Vero cells (Figure 1B). In contrast, growth of the virus that contained 44 wt C gene codons (or less) was significantly attenuated compared to ZIKV-NS3m (Figure 1B; *p* < 0.0001, 2-way ANOVA). This indicated that 3′-terminus of C gene regulatory region is located between codons 44 and 73. Using m-fold RNA secondary structure prediction analysis [30], we identified several potential stem-loop (SL) elements located within 5′ non-coding region (NCR) and C gene of ZIKV (Figure 2A). The region between codons 44 and 73 of C gene is occupied by two RNA elements, SL6 and SL7. The SL7 (the most 3′-distant RNA secondary structure) is unlikely to play a significant role in ZIKV replication in Vero cells, since this structure was disrupted in the virus C73 (Figure 1 and Figure 2A).

To precisely delineate sequence of the C gene regulatory region, we used the strategy of capsid gene duplication, which has been employed previously for mapping *cis*-acting elements of Langat virus (LGTV) and Dengue type 2 viruses (DEN2) [31,32]. For that, the regulatory and structural functions of the C gene of ZIKV-NS3m were separated and expressed from two different sequence elements in the virus C67-Ubi (Figure 2B). The upstream copy of C gene in C67-Ubi was encoded by the 5′-terminal 201 nts (67 AA) of the ZIKV wt C gene sequence. It contains the *cis*-acting elements involved in the ZIKV replication. The translation of functional C protein from this copy of C gene was ablated by insertion of a single adenine residue after the fourth codon, shifting open reading frame (ORF) of viral polyprotein. Insertion of adenine residue at this position exerts a minimal effect on ZIKV RNA secondary structure, as verified by m-fold prediction analysis, and does not generate stop codons in the downstream sequence. The downstream copy of C gene (C opt) was modified by the introduction of synonymous substitutions in each of AA codons. This disrupts all *cis*-acting elements located in C opt while preserving the correct AA sequence, which is necessary for formation of functional C protein. The release of N-terminus of C protein translated from C opt was ensured by insertion of a Ubiquitin gene upstream of C opt sequence. To restore a correct open reading frame orientation of Ubiquitin gene, and all downstream sequences of ZIKV ORF with respect to the initiation codon AUG located in the SL2, we inserted the target sequence (20 nt) for cellular microRNA-124-3p (mir-124(T)) between the upstream copy of C gene and Ubiquitin. MicroRNA-124-3 is expressed exclusively in the central nervous system [33]. Therefore, insertion of a target sequence for this miRNA into the genome of C67-Ubi does not affect replication of the virus in the kidney-derived Vero cells [31], while separating *cis*-acting elements of ZIKV from Ubiquitin gene. In addition, introduction of mir-124(T) was done to demonstrate utility/suitability of the selected site for insertion of different targets for cellular microRNAs into ZIKV genome. Subsequently, clone C67-Ubi was used as a template to generate a panel of viruses featuring gradual deletions of the upstream copy of C gene from its 3′ terminus (Figure 2B). Compared to the C67-Ubi virus, a significant reduction in the growth rate in Vero cells (Figure 2C; *p* < 0.0001, 2-way ANOVA) and reduction in plaque size morphology (Figure 2D) was observed for viruses C40-Ubi and C45-Ubi, but not for the viruses that contained 50 (or more) codons of the C gene. This data indicate that 3′-terminus of the C gene regulatory region is located between codons 45 and 50. The sequence of the first 50 codons (150 nts) of wt C gene was designated as a complete ZIKV C gene regulatory region that would be used in the experiments aimed at the development of ZIKV clones capable of stably expressing heterologous genes of interest.

### 3.2. Development of ZIKV Clones Expressing nLuc Gene in the Duplicated Capsid Gene Region (dCGR) and Duplicated E/NS1 Junction Region

To compare efficiency of heterologous gene expression and the insert stability among the different approaches for insertion of foreign gene(s) into ZIKV genome, we selected an infectious clone ZIKV-ICD of the strain Paraiba_01/2015 [23] as a common genetic background for clone construction. The nLuc gene was chosen as a common bioluminescent reporter for the insertion into all constructs due to its relatively small size (171 aa) and high signal intensity [34].

**Figure 3:**
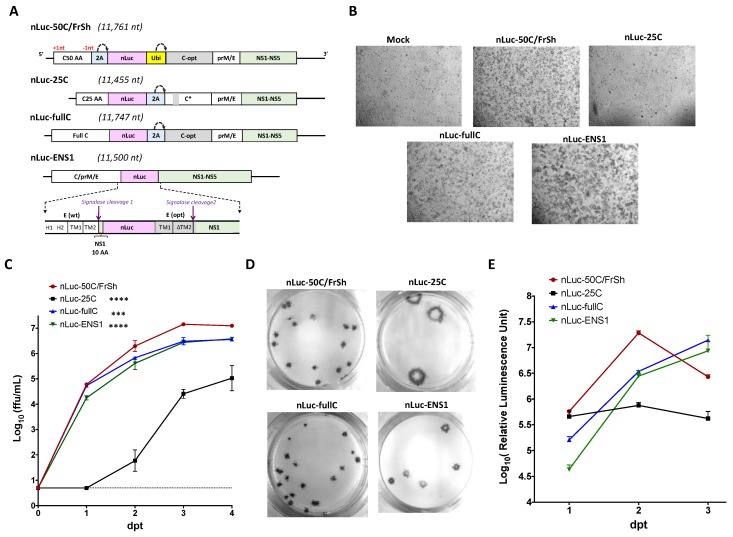
Construction and characterization of nLuc-carrying viruses in Vero cells. (**A**) Genetic organization of nLuc-containing viruses. ORF shifting and restoring mutations are highlighted as +1nt and −1nt, respectively. 2A: 2A protease sequence from FMDV; Ubi: ubiquitin sequence; gray boxes highlight codon-optimized sequences in the C and E genes of ZIKV. C* in nLuc-25C is a C opt gene with mutations in 14–17 AA codons. Dotted arrows represent the sites of cleavage by 2A protease or ubiquitin. (**B**) Microscopic evaluation (at 40× magnification) of CPE in Vero cells monolayer observed on day 6 after plasmid DNA transfection (dpt). (**C**) Growth kinetics of nLuc-carrying viruses in Vero cells after plasmid DNA transfection. Mean virus titer ± standard deviations in the samples that were collected daily from duplicate flasks was determined by titration on Vero cells. Dotted line represents limit of detection of the FFA (0.7 Log_10_(ffu/mL)). Differences between growth kinetics of nLuc-50C/FrSh and those of the other three constructs were compared using two-way ANOVA (**** *p* < 0.0001; *** *p* < 0.001). (**D**) Plaque morphology of nLuc-carrying viruses in Vero cell monolayer as revealed by immunostaining at 5 dpi. (**E**) Kinetics of luciferase activity following plasmid DNA transfection into Vero cells.

Since the virus C50-Ubi contains the complete ZIKV C gene regulatory region (Figure 2), it was selected as a genetic template for insertion of nLuc gene in the dCGR. For that, we replaced the sequence for mir-124-3p target in the C50-Ubi (Figure 2B) with that of nLuc gene (Figure 3A), followed by insertion of the resulted dCGR into the genetic background of ZIKV-ICD. To catalyze proteolytic release of an authentic N-terminus of nLuc protein, a single copy of 2A protease from FMDV was inserted upstream of nLuc gene in a manner that restores the proper ORF alignment, which was disrupted by a single nt insertion after the fourth codon of C gene. In the resultant construct nLuc-50C/FrSh, possible recombination between two copies of C gene should lead to formation of non-viable virus due to the presence of reading frame shift and codon optimizing mutations in the upstream and downstream copies of C gene sequences, respectively (Figure 3A).

We used the background of ZIKV-ICD to construct two infectious clones (designated nLuc-25C and nLuc-fullC) to reproduce the two approaches previously described for heterologous gene expression from ZIKV genome (Figure 3A). The nLuc-fullC virus, generated using strategy described by Mutso et al. (see construct ZIKV-NanoLuc in [17]) contained nLuc gene inserted between two full-length copies of the C gene, of which the downstream copy was codon-optimized. For the nLuc-25C virus, which represents strategy described by Shan et al. (see construct ZIKV-RLuc in [18]), we preserved only 25 AA codons of the upstream copy of wt C gene, followed by nLuc and the full-length C gene. In contrast to nLuc-fullC or nLuc-50C/FrSh viruses, in the nLuc-25C only codons 14–17 in the downstream copy of C gene were mutated by synonymous substitutions (Figure 3A). In both nLuc-25C and nLuc-fullC constructs, release of downstream copy of C gene from nLuc was catalyzed by 2A protease from FMDV.

It was shown that several flaviviruses can also stably express heterologous genes inserted into the duplicated E/NS1 junction region instead of dCGR [20,21]. To explore if a similar approach can be used to drive gene expression in the context of ZIKV, we inserted nLuc after the tenth codon of NS1 protein of ZIKV-ICD preserving signalase cleavage site between E and NS1 proteins. To ensure proper membrane orientation and processing of ZIKV polyprotein, we introduced a second copy of the signalase cleavage site by inserting after nLuc gene a codon-optimized copy of the TM1 and TM2 regions of E gene, followed by a full-length NS1 gene (see nLuc-ENS1 in Figure 3A). The virus produced by transfection of the resultant infectious clone into Vero cells was harvested and sequenced. We observed that the recovered virus retained the full sequence of the upstream TM1/TM2 but accumulated a deletion of three codons encoding Val-Leu-Ile amino acids (aa 494–496 in the original ZIKV E protein) in the downstream copy of TM2 region (see ∆TM2 in Figure 3A). Since this deletion increases replicative fitness of the virus, and it also reduces the probability of possible recombination event between the two copies of the E/NS1 junction region, we retained it for the final iteration of the nLuc-ENS1 virus.

### 3.3. Characterization of the nLuc-Carrying Viruses In Vero Cells

Infectious nLuc-carrying viruses were rescued by transfection of plasmid DNAs into duplicate 12.5 cm^2^ flasks of Vero cells. The nLuc-ENS1was the most cytopathic of the four luciferase-carrying viruses, while nLuc-25C did not produce observable CPE on day 6 following DNA transfection (Figure 3B). None of the viruses caused CPE strong enough to develop detectable plaques on Vero cell monolayer, and virus titers were determined by an FFA using ZIKV reactive antibodies. Among the four nLuc-carrying viruses, the nLuc-50C/FrSh replicated most efficiently in Vero cells, reaching titer of ~7.0 log (ffu/mL) by 3 dpt (Figure 3C). Growth of nLuc-fullC and nLuc-ENS1 was similar and moderately attenuated compared to nLuc-50C/FrSh (Figure 3C; *p* < 0.001, 2-way ANOVA). The nLuc-25C was the most attenuated construct that attained the lowest titer among the four viruses (Figure 3C). Analysis of focus morphology, performed at 5 dpt, showed that foci produced by nLuc-25C were considerably larger and more diffused than those generated by other viruses (Figure 3D).

To compare kinetics of nLuc expression, Vero cells in 24-well plate were transfected with plasmid DNAs encoding each of the four nLuc-carrying viruses. The nLuc activity was measured daily in Vero cell lysates from the duplicate wells. Cell transfected with nLuc-50C/FrSh construct demonstrated fastest kinetics of nLuc accumulation, reaching maximum nLuc expression one day earlier compared to nLuc-fullC and nLuc-ENS1viruses (Figure 3E). Following plasmid DNA transfection with viral constructs, the nLuc gene can be translated from two distinct mRNAs: 1—the mRNA transcribed directly from the transfected plasmid in the nucleus from CMV promoter; and 2—from positive sense genomic RNA, which was synthesized in the cytoplasm by viral polymerase complex during viral replication. The absence of detectable increase in nLuc activity after 1 dpt for nLuc-25C virus (Figure 3E) strongly suggested that nLuc was translated from CMV promoter-derived mRNA rather than from viral RNA synthesized in the cytoplasm.

### 3.4. Assessment of nLuc Insert Stability in Viral Genomes during Repeated Passages in Cell Culture

**Figure 4:**
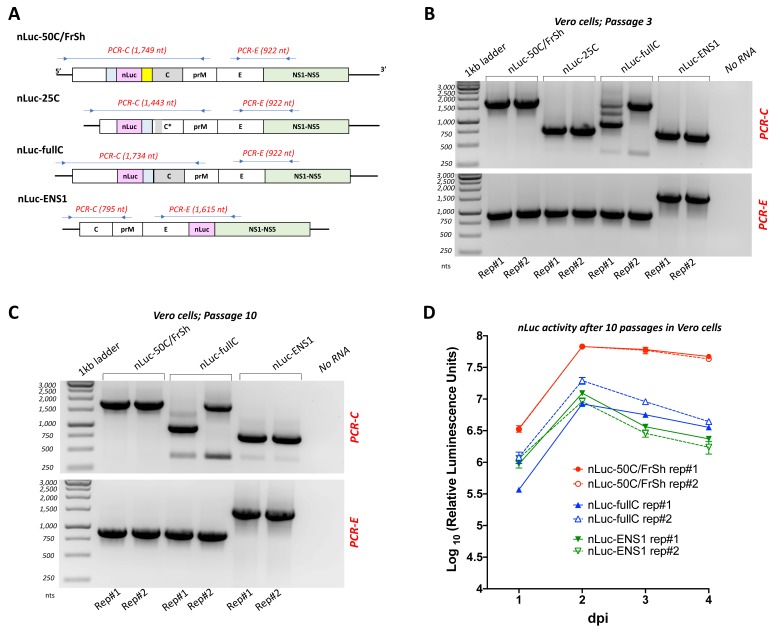
Evaluation of the insert stability of recombinant nLuc-carrying viruses. To compare genome stability of ZIKV-carrying nLuc, blind passaging was performed for all four viruses in 12.5 cm^2^ flasks of Vero cells in duplicates, and RT-PCR reactions targeting the regions of nLuc insertion were carried out at multiple points during the experiment followed by sequencing. (**A**) Schematic representation of the positions of primers (represented by arrows) and expected lengths of corresponding RT-PCR fragments. (**B**,**C**) Agarose gel electrophoreses of RT-PCR fragments produced using viral RNA extracted from duplicate flasks of Vero cells (Rep#1 and Rep#2) after passage three (panel B) and ten (panel C). For each virus, two RT-PCR reactions were carried out (PCR-C and PCR-E, indicated in red), amplifying the region of nLuc gene insertion and selected region of ZIKV genome. Amplification of the viral regions that do not contain nLuc insertion serves as control of the expected band sizes after a complete deletion of the heterologous sequences in the nLuc-carrying viruses. Negative template control was included in each experiment and is shown in No RNA lane. Passaging of nLuc-25C was terminated after the third passage due to complete loss of nLuc insertion. (**D**) Kinetics of luciferase activity in Vero cells. Viruses recovered after the tenth passage in Vero cells were used for infection of Vero cells in 24-well plates at an MOI of 0.1. Measurements of nLuc activity in cell lysates were performed daily and data expressed as mean of standardized luciferase units ± standard deviations for the duplicate wells of infected Vero cells.

Comparatively slower growth in association with large focus size phenotype of the nLuc-25C virus (Figure 3C,D) might be associated with insert instability resulting in deletion of the nLuc gene from the viral genome. To compare the stability of the inserts among the four approaches for heterologous gene expression in ZIKV, we performed repeated blind passages of all four luciferase-carrying viruses in Vero cells, followed by RT-PCR and sequencing analysis (Figure 4). The region of nLuc gene insertion remained stable in nLuc-50C/FrSh and nLuc-ENS1 for at least ten blind passages (Figure 4B,C). Both viruses recovered after ten passages from each of the two replicates were capable of supporting high nLuc expression in Vero cells, although luciferase activity was ~3–10 folds lower in the cells infected with nLuc-ENS1 viruses as compared to that of nLuc-50C/FrSh (Figure 4D). In contrast, nLuc-25C had completely lost nLuc insertion in both replicates after only three passages in Vero cells (Figure 3B), which was verified by sequencing analysis, and was not used for further characterization. Interestingly, in one of the two experimental replicates of nLuc-fullC virus, the partial ejection of the heterologous sequence was detected as early as the third Vero cell passage, which is reflected by appearance of multiple bands in the RT-PCR reaction (Figure 4B). Sequencing analysis of the predominant RT-PCR band amplified from nLuc-fullC virus after the tenth passage of this replicate in Vero cells showed that the virus did not revert to wt genome configuration. Instead, it selected a deletion that started after 41 nt of the nLuc gene and ended at the 3′-terminal part of the second (codon-optimized) copy of C gene (Figure S2). In contrast, the nLuc-fullC virus from the second experimental replicate remained stable for ten passages in Vero cells (Figure 4C) and was capable of supporting substantially higher nLuc expression, as compared to the virus collected from the first experimental replicate (Figure 4C).

### 3.5. Construction of eGFP-Expressing ZIKV and Characterization of Reporter Viruses in Cell Culture

Since approaches for heterologous gene expression that preserve either the whole C or the first 25 aa of C gene at 5′ end of the genome demonstrated a high tendency for genetic instability, they were excluded from further characterization. In contrast, both nLuc-ENS1 and nLuc-50C/FrSh viruses remained stable in Vero cells (Figure 4) for ten passages and were chosen for subsequent analysis. 

To test whether the developed approaches are suitable for gene expression of other functional heterologous proteins, we replaced nLuc gene in the nLuc-ENS1 and nLuc-50C/FrSh viruses with that of eGFP. Transfection of Vero cells with construct carrying eGFP in the dCGR (designated GFP-50C/FrSh) resulted in detectable levels of eGFP expression. Repeated blind passaging of the GFP-50C/FrSh virus in Vero cells showed that the level of eGFP expression remained unchanged in the course of the experiment (Figure S3A). After the tenth passage, eGFP gene insertion cassette was intact in the viral genome as confirmed by RT-PCR and sequencing analysis, indicating high genetic stability of the virus (Figure S3B). However, two minor RT-PCR bands of shorter length were also detectable in agarose gel, suggesting appearance of ZIKV mutants that might have accumulated deletions in the eGFP gene during passaging (Figure S3B). In contrast to GFP-50C/FrSh, insertion of eGFP into the duplicated E/NS1 junction region (designated GFP-ENS1) of ZIKV did not cause detectable fluorescence in Vero cells after plasmid DNA transfection. This indicates that duplicated E/NS1 site is more restrictive for the functional expression of the heterologous genes of interest in the context of ZIKV genome as compared to the dCGR. Considering this and the fact that nLuc-ENS1 attained significantly lower titer in Vero cells compared to nLuc-50C/FrSh (Figure 3C), we excluded viruses generated using the approach of E/NS1 region duplication from further characterization.

**Figure 5:**
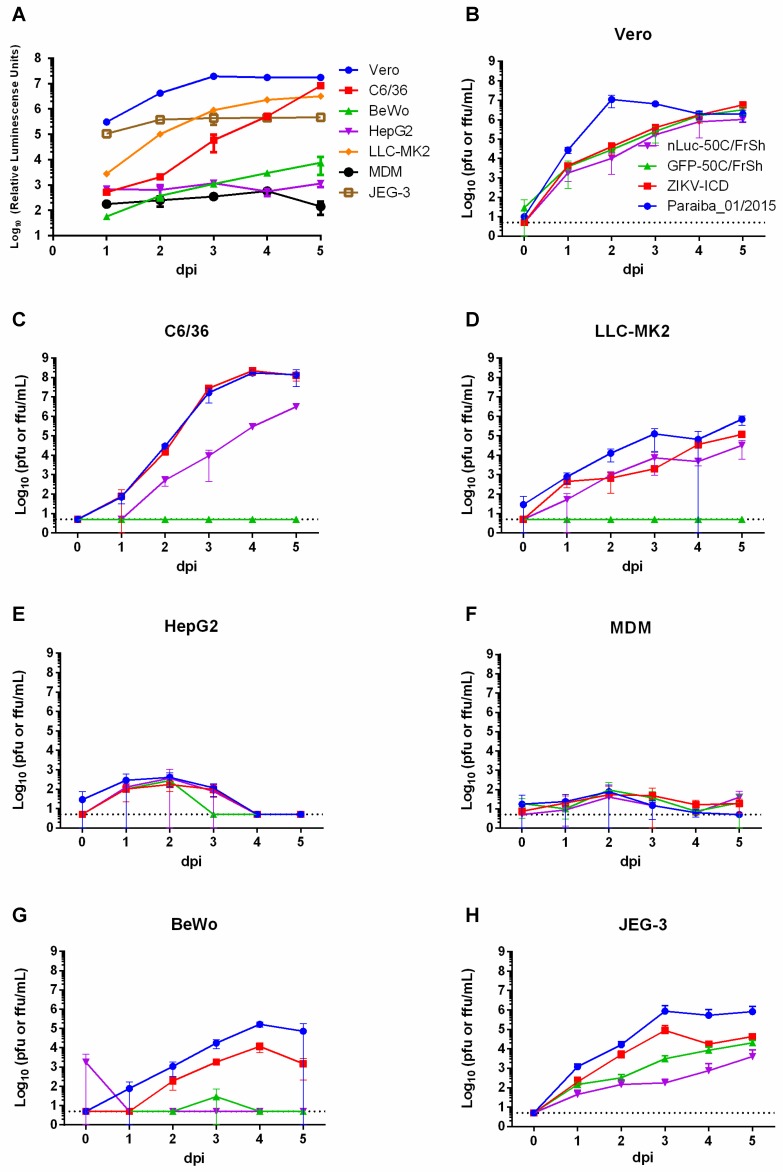
Characterization of reporter-carrying viruses in different cell lines. (**A**) Various cell lines of primate, human, and mosquito origin and human primary monocyte derived macrophages (MDM) were infected with nLuc-50C/FrSh in duplicates at an MOI of 0.01 in 24-well plates. Luciferase activity in cell lysates was measured daily for five days. (**B**–**H**) Growth kinetics of nLuc-50C/FrSh, GFPc-50C/FrSh, parental Paraiba_01/2015, and recombinant ZIKV-ICD viruses (colors representing each virus are defined in panel **B**) in Vero (**B**), C6/36 (**C**), LLC-MK2 (**D**), HepG2 (**E**), MDM (**F**), BeWo (**G**), JEG-3 (**H**). Cells were infected with each virus at a MOI of 0.01 in 12.5 cm^2^ flasks in duplicates. Aliquots were taken every day for five days after infection, and FFA or PFA was performed to determine virus titers. Results are presented as mean values of two biological replicates with SD shown as error bars. Dotted line represents limit of detection of the assay (0.7 Log_10_(pfu or ffu/mL)).

To characterize the reporter-carrying viruses nLuc-50C/FrSh and GFP-50C/FrSh in cell culture and compare them with a parental ZIKV-ICD clone-derived virus (as well as with natural ZIKV Paraiba_01/2015 isolate, which was used for ZIKV-ICD construction), various cell cultures were infected, and growth curves were generated with data collected for up to 5 days (Figure 5B–H). For the nLuc-50C/FrSh, luciferase activities were determined every day for 5 days post-infection (Figure 5A), and eGFP expression was observed in cells infected with GFP-50C/FrSh virus. Growth kinetics of the recombinant viruses were almost indistinguishable in Vero cells (Figure 5B). While parental virus reached higher titers earlier in the infection, the difference disappeared by day 4. In HepG2 and MDMs cells (Figure 5E,F), none of the viruses were able to produce high infectious titers; however, it is worth noting that the primary cell culture was produced from a single blood donor, therefore this result might not be representative. Unlike nLuc-50C/FrSh virus, the GFP-50C/FrSh was unable to achieve productive replication in C6/36 and LLC-MK2 cells (Figure 5C,D). It is possible that stronger attenuation of the GFP-50C/FrSh in these cell lines is due to the larger size of eGFP gene as compared to the nLuc gene (717nt vs. 513nt). Parental virus and ZIKV-ICD exhibited the same growth characteristics in C6/36 cells, whereas nLuc-50C/FrSh was attenuated in this cell line. In all other cell lines, parental virus attained higher titers compared to the nLuc-50C/FrSh and GFP-50C/FrSh viruses. Interestingly, BeWo cells, unlike JEG-3, were unable to maintain replication of the reporter-carrying viruses, despite the fact that both of these cell lines are of placental origin (Figure 5G,H).

## 4. Discussion

In this study, we characterized two novel approaches for expression of heterologous genes of interest in the context of ZIKV genome. Both approaches rely on duplication of a specific region of ZIKV genome (CGR and E/NS1), which allows uninterrupted translation and correct processing of viral proteins from a single ORF. Compared to previously described strategies for heterologous gene expression [17,18], both approaches described here were associated with increased stability of the inserted genes during prolonged passaging of the viruses in cell culture. However, we observed that, compared to E/NS1 approach, ZIKV clones generated using dCGR strategy attained higher titers in cell culture and were associated with a higher expression of the heterologous gene in the infected cells. Moreover, compared to E/NS1 duplication, the dCGR strategy appeared to be less restrictive regarding the nature of inserted genes (more flexible), potentially permitting generation of ZIKV clones capable of productively expressing a greater number of heterologous genes of interest.

The 5′ region of the flavivirus genome (which includes C gene regulatory elements) regulates viral replication through genome cyclization and other mechanisms [35]. To develop viruses expressing nLuc and eGFP in the dCGR, we mapped a complete regulatory region located in the C gene of ZIKV (Figure 1 and Figure 2). It appears that ZIKV regulatory elements are confined to the first 50 codons of C gene. This region is larger than that of DENV-2 (~35 codons [27]) and is similar in length to the C gene replication promoter of LGTV (48 codons [31]). Similar to the C gene regulatory region of LGTV (see [31,36]), sequence folding analysis predicted existence of a well-defined stem-loop structure located at the 3′ end of ZIKV C gene regulatory region (see SL6 in Figure 2). For LGTV, it was shown that the sequence located at the tip of the 3′-terminal stem-loop structure of C gene regulatory region engages in complementary interaction with nucleotides located at the tip of another stem-loop structure located at the 3′NCR of the virus. This so-called ‘kissing loop’ interaction helps to stabilize pan-handle RNA structure formed during LGTV genome cyclization. Interestingly, the sequence located at the tip of SL6 of ZIKV C gene regulatory region has four complementary motifs located in different regions of 3′NCR of the virus, suggesting potential ‘kissing’ interactions between these parts of ZIKV genome. Future studies will delineate the role of SL6 (and other sequences located within C gene regulatory region) in ZIKV life cycle and correlate resulting findings with unique aspects of ZIKV pathogenesis. The ZIKV clones with dCGR, expressing nLuc or other reporter genes, will be valuable experimental tools to address these questions.

The virus nLuc-25C, which represents strategy described by [18], appears to contain only 5′-terminal half of the complete C gene regulatory region and lacks codons 26–50, which are essential for ZIKV replication. This may explain attenuated growth and rapid reversion of the virus to wt genome configuration during passaging in Vero cells. In contrast, the complete C gene regulatory region was preserved in the nLuc-fullC virus. Nevertheless, the growth of this virus was also significantly attenuated compared to nLuc-50C/FrSh (Figure 3). Genomes of nLuc-fullC and nLuc-50C/FrSh viruses differ by several sequence elements, each of which can contribute to the difference in growth rates of these viruses. We speculate that one of the attenuating factors for nLuc-fullC might be the stoichiometric imbalance between the amounts of C protein produced in the infected cell compared to the levels of ZIKV RNAs and other ZIKV genes products. In the nLuc-fullC virus, the capsid protein can be expressed from two full copies of C gene, as opposed to only one copy in wt ZIKV. This imbalance can promote premature encapsidation of the genomic RNAs, sequestering it from entering genome replication cycle. In agreement with this explanation is the fact that the escape deletion that occurred in the nLuc-fullC virus in Vero cells did not restore wt genome configuration, but rather eliminated most of the downstream copy of the C gene, restoring appropriate stoichiometry of viral proteins (Figure S3). In contrast, there is only one copy of C gene in the genome of nLuc-50C/FrSh that is responsible for the synthesis of the C protein (see C opt in Figure 3A). This therefore would lead to a correct (wt -like) stoichiometry of all ZIKV proteins in the nLuc-50C/FrSh infected cells. Considering that engineered disbalance in stoichiometry of viral gene products is being considered as mean of rational attenuation of several flaviviruses [37,38], understanding the effects of additional copy of C gene on ZIKV fitness might have direct impact on vaccine research studies.

We speculate that the superior stability of nLuc-50C/FrSh (and GFP-50C/FrSh) observed in our experiments is due to synergistic effects of codon optimizing and the frame shifting mutations inserted into separate copies of duplicated C gene. The codon optimization prevents homologous recombination from occurring between the two copies of the capsid gene, whilst the frame shifting ensures that even if recombination does occur, no functional capsid protein is produced. It is also possible that insertion of frame shifting mutation into upstream copy of C gene increases replicative fitness of ZIKV with dCGR by a mechanism similar to what was proposed for LGTV with dCGR [31]. For instance, frame shifting ensures that truncated capsid protein cannot be translated and would not interact or interfere with the function of full-length copy of C protein translated from codon optimized copy of C gene, or with genomic and/or antigenomic viral RNAs.

Viruses containing reporter genes within their genomes are a useful tool for detection and/or quantification of viral replication. Fluorescent reporter-carrying viruses can be directly visualized to investigate ZIKV tropism in cell culture and in vivo. Since nLuc expression can be detected quantitatively in animal organs and tissues not requiring euthanasia, this makes the Luc clones a relevant tool for studying kinetics of ZIKV infection and dissemination within individual animal or between different animals in the studies of viral transmission. The utility of the dCGR approach extends beyond investigations of viral tropism and dynamics of dissemination involving in vivo fluorescence and/or bioluminescence imaging studies; the same region can be used to insert heterologous sequences other than reporter genes. We have shown that ZIKV can tolerate a relatively large sequence insertions (at least 717 nts in case of eGFP) between the two copies of capsid gene, making our findings relevant to broad areas of biomedical research, including vaccine development, discovery of antiviral compounds, and pathogen inactivation techniques or development of diagnostic assays. For instance, development of ZIKV vaccine candidates created by inserting targets for host microRNAs specific for certain tissues into dCGR has already been reported [24].

Although recombinant bioluminescent constructs created for several members of family *Flaviviridae* have been used to investigate viral replication dynamics and pathogenicity in animal models [15,16,39], previously described bioluminescent ZIKV systems have not been applied yet to studying virus tropism in live animals [17,18]. We demonstrated that among the four tested viruses expressing nLuc gene, the nLuc-50C/FrSh virus attained the highest titer in Vero cells, which was associated with the highest level of nLuc gene expression in the infected cells. The virus remained stable after at least ten passages in Vero cells, suggesting that the approach based on partial capsid gene duplication might be one of the most suitable strategies for construction of reporter ZIKV for in vivo imaging experiments and other purposes. Further studies employing animal models of ZIKV infection are needed to evaluate performance of the dCGR reporter viruses.

## 5. Conclusions

In this study, we mapped the sequence of the complete ZIKV regulatory region within C gene and compared existing and newly developed approaches for heterologous gene expression from ZIKV genome. The results of our study demonstrate the suitability of the dCGR strategy for development of stable replication-competent ZIKV infectious clones expressing specific sequence of interest. The convenience and versatility of eGFP and nLuc detection protocols make the nLuc-50C/FrSh and GFP-50C/FrSh clones relevant tools for studying kinetics of ZIKV infection in cell culture and in vivo. Further tests of these viruses in animals are warranted to evaluate their utility for studying viral tropism and dissemination in vivo.

## Supplementary Materials

The following are available online at https://www.mdpi.com/1999-4915/12/1/61/s1, Figure S1: Genetic organization and characterization of plasmids encoding nLuc-carrying cDNA ZIKV infectious clones, Figure S2: Analysis of deletion in the genome of nLuc-fullC virus observed after ten passages in Vero cells, Figure S3: Stability of GFP-50C/FrSh during repeated passaging in Vero cells.
