# Supplementary material for: Novel Approach for Insertion of Heterologous Sequences into Full-Length ZIKV Genome Results in Superior Level of Gene Expression and Insert Stability
Source: Viruses. 2020 Jan 3;12(1):61. doi: 10.3390/v12010061 (PMC7019263; doi:10.3390/v12010061)
Supplement: Supplementary file 1 [file viruses-12-00061-s001.pdf]

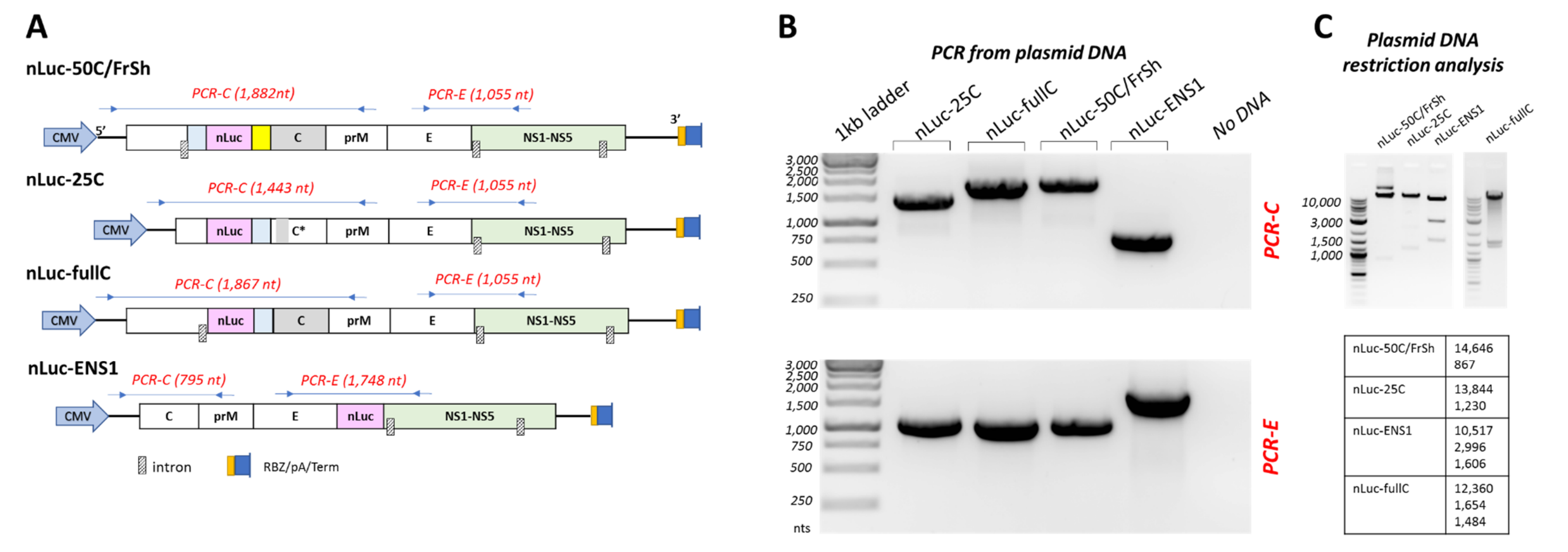

nLuc-50C/FrSh	14,646<br>867
nLuc-25C	13,844<br>1,230
nLuc-ENS1	10,517<br>2,996<br>1,606
nLuc-fullC	12,360<br>1,654<br>1,484

Figure S1. Genetic organization and characterization of plasmids encoding the nLuc-carrying clones. **A.** Schematic representation of intron positions in the plasmids and expected lengths of corresponding PCR fragments. **B.** Agarose gel electrophoresis of PCR fragments produced from plasmid DNA using primers depicted in the pannel **A**. **C.** Agarose gel electrophoresis of DNA fragments produced after digestion of plasmid DNA with several restriction endonucleases. Expected lengths of DNA fragments (nts) after digestion of the plasmids are summarized in the table located at the bottom of the panel **C**.

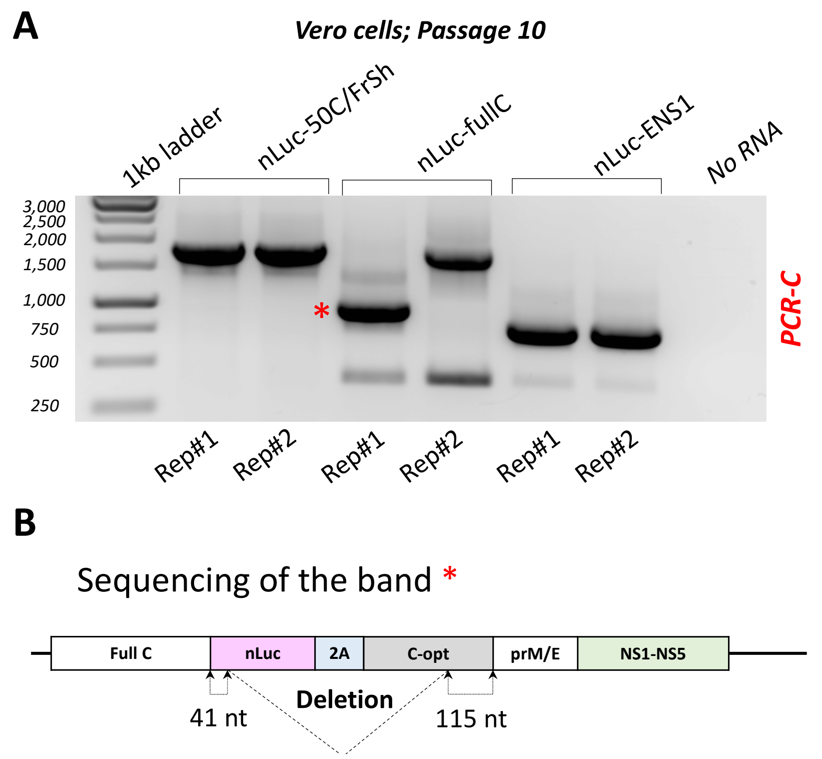

Figure S2. Analysis of deletion in the genome of nLuc-fullC virus observed after 10 passages in Vero cells.

A. Agarose gel electrophoresis of RT-PCR fragments produced using RNA extracted from duplicate flasks of Vero cells (Rep#1 and Rep#2) depicted in the **Fig. 4C** of the manuscript. Whilst genomic regions carrying nLuc gene in nLuc-50C/FrSh and nLuc-ENS1 viruses remained intact, one of the two replicates of nLuc-fullC (highlighted with asterisk) appeared to have lost the insertion. **B**. Sequencing of the PCR product indicated on panel **A** by the asterisk revealed a partial deletion of nLuc-expressing cassette (highlighted with dashed arrows). The deletion preserved 5'-terminal 41 nts of the nLuc gene, which are followed by 115 3'-terminal nts of optimized C gene. Collectively, these sequences would be translated into a 52 AA appendage at the end of the upstream full-length copy of C protein.

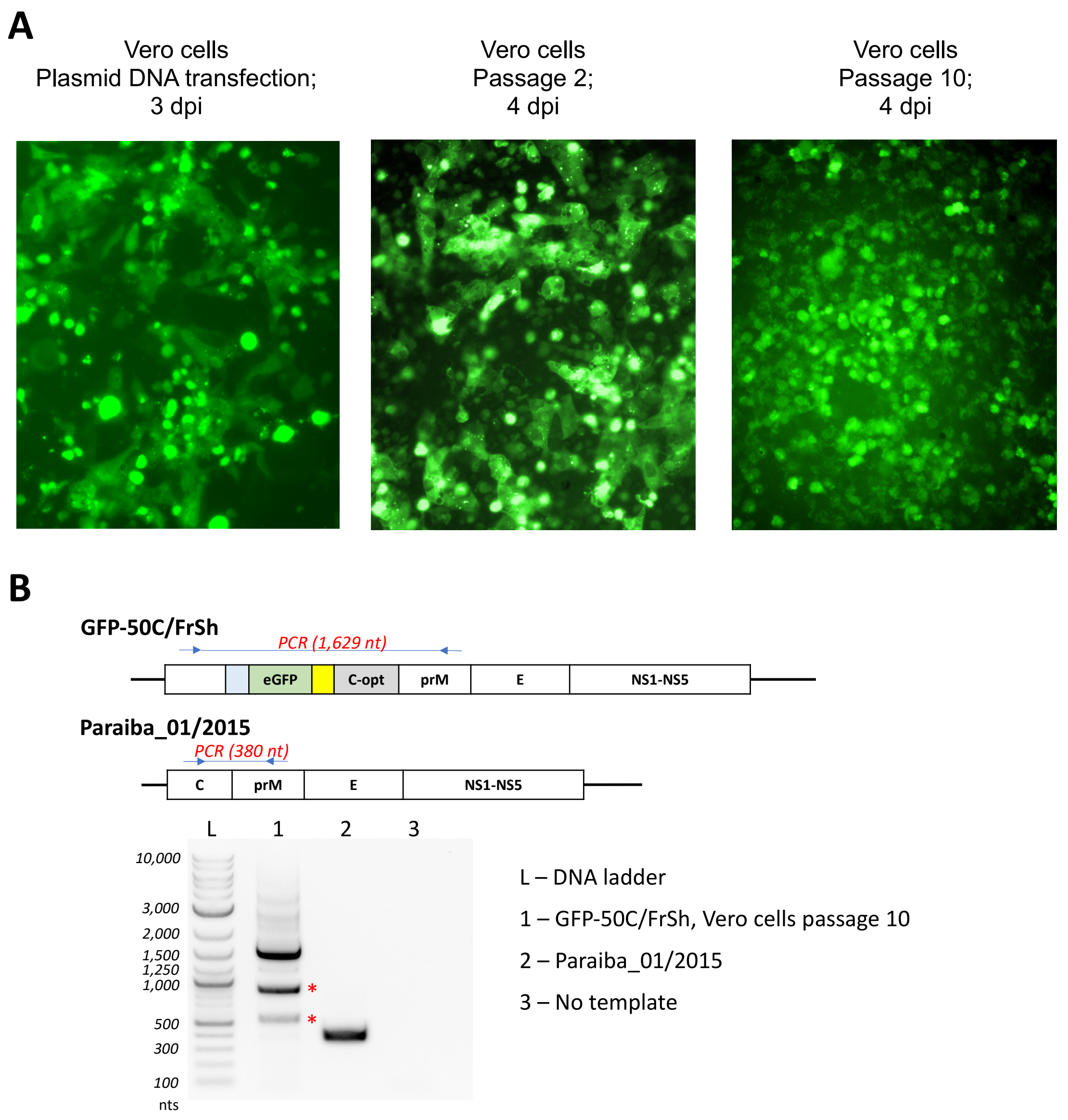

Figure S3. Stability of GFP-50C/FrSh during repeated passaging in Vero cells.
A. Microscopic evaluation of an eGFP expression in Vero cells transfected with plasmid DNA of GFP-50C/FrSh or infected with GFP-50C/FrSh virus collected after the 2nd or 10th passage in Vero cells. **B**. Agarose gel electrophoresis of RT-PCR fragments produced using RNA extracted from Vero cell supernatants after passage 10 of GFP-50C/FrSh. ZIKV Paraiba_01/2015 was used as positive control to estimate the length of the RT-PCR fragment produced after deletion of all heterologous sequences from GFP-50C/FrSh.
